# The effects of aging and gestational month on uteroplacental vascular perfusion, and umbilical artery hemodynamics in pregnant jennies

**DOI:** 10.1186/s12917-022-03499-8

**Published:** 2022-11-15

**Authors:** Elshymaa A. Abdelnaby, Ibrahim A. Emam, Hossam R. El-Sherbiny, Aya M. Fadl

**Affiliations:** 1 Theriogenology Department, Faculty of Veterinary Medicine, Cairo University, Giza Square, Giza, 12211 Egypt; 2 Department of Surgery, Anesthesiology and Radiology, Faculty of Veterinary Medicine, Cairo University, Giza, Egypt

**Keywords:** Donkey, Doppler, Umbilical, Middle uterine artery, Pulsatility

## Abstract

This study aimed to correlate the pulsed wave spectral indices of the middle uterine artery at both sides with placental development in jenny within mid-late pregnancies, and establish umbilical Doppler values for different ages and different gestational months. Twenty Equus Asinus pregnant jennies 260–450 kg (average, 320 ± 10 kg) were examined from 5 to 9 months of pregnancy with different ages (4–14 years). Monthly B-mode ultrasound examination was performed on both the combined thickness of the uterus and placenta (CTUP; mm) and umbilical artery cross-sectional diameter, and Doppler mode examination was performed on both the middle uterine (MUA at right [R] and left [L] sides) and umbilical arteries to measure both Doppler indices that expressed by resistance (RI) and pulsatility indices (PI), and blood flow rate. CTUP was elevated within pregnancy time at different ages (*P* < 0.05). L. PI was significantly declined throughout different ages (*P* < 0.05), but this declining trend was not observed in L. RI. The L. blood flow rate (R; bpm) was elevated among different ages and different months (*P* < 0.05). Both RI and PI were significantly decreased from 5 to 9 month of gestation period in jennies (*P* < 0.05).. The umbilical arteries cross-sectional diameter (Umb A; mm), was elevated among different ages and different months, while both Doppler indices were declined. A positive correlation was found (between both Doppler indices of both umbilical and uterine arteries *P* < 0.001). There was elevated vascular perfusion in uterine and umbilical arteries associated with reduced both Doppler indices along the course of pregnancy at different ages.

## Introduction

In recent years, the advancement of assisted reproductive technology has set new standards in equine reproductive overall performance, resulting in extraordinary achievements in conception rates for subfertile/infertile populations. In contrast, little progress has been made in the field of monitoring pregnancy, and efforts to obtain a better understanding of physiological features during pregnancy in jenny remain necessary [1]. The growth in fetal size with the uterine environment in cows and mares in the last trimester is related to a growing demand for vitamins and oxygen ensured via way of means of growth in the uterine and fetal perfusions [2, 3]. The uterine arteries are especially responsible for the maternal part, while the umbilical arteries supply the fetal part [4, 5]. Uterine vascularization can be assessed using the pulsatility index (PI) and resistance index (RI) of the uterine arteries, which are quantitative measures of vascular perfusion of the reproductive structures and are not motivated by the Doppler angle [6–8]. Blood flow is normally measured via both Doppler indices (PI and RI). The latter reveals a negative relationship with vascular perfusion, as the growth in resistance shows a lowering-vascular perfusion [9–12]. Therefore, any alterations in vascular perfusion generally occur due to poor placental performance, fetal growth abnormalities, and hypoxia [13, 14]. In the current study, the thickness of the uterus and placenta (CTUP/mm) was measured in order to determine the functional status of the placenta, as previously reported in mares [15, 16], and donkeys [17].

Doppler studies of jenny gestational vessels, such as middle uterine and umbilical arteries, could give a prediction value of the gestation outcome [18]. It was reported that there was a monthly elevation of the uterine artery that led to a strong increase in blood flow rate and a marked decline in the RI during the first 8 months of pregnancy in buffalos [19].In addition, in mare the same declination in both Doppler indices was observed within gestational months [16], as this could be due to the presence of endothelial factors that were associated with the marked decline in both Doppler indices and related to arterial hypertension [17]. However, a study reported that uterine blood flow did not differ between pregnant and nonpregnant mares [20]. In comparison to others, the umbilical cord information of the jennie fetus is less well known, as there were no references to data supporting the pregnancy profile and fetus formulation [17, 21] that recognized many pregnancy problems at different gestational stages. Therefore, we aimed to correlate the pulsed wave spectral indices of the middle uterine artery at the right and left sides with placental development in form of the CTUP in jenny within advanced gestation, and to establish umbilical Doppler reference values for different ages and different gestational months.

## Materials and methods

### Ethical approval

All procedures were conducted on pregnant animals following the international ethical committee for animal use protocol (Vet CU 23,052,022,461) at the Faculty of Veterinary Medicine, Cairo University.

### Animals and management

Twenty multiparous pregnant jennies were assessed in this current study between 4 and 14 years of age. Animals were kept in a large animal farm at the Department of Surgery, Cairo University. Animals were used under the permission of the institution. All females weighed between 260–450 kg (average, 320 ± 10 kg) with a number 3 body condition score [22]. All animals were routinely vaccinated against diseases; in addition, clinical examination was performed for all animals. Females were bred naturally with an excellent fertile stallion, all mares were with a normal course of pregnancy without any complications such as (placental separation, abortion, unusual activity, and premature birth pain).

### Ultrasound assessment

All jennies were examined at 5–9 gestational months using linear array rectal probe (ExaGo, France) supplied with frequency (5–7.5 MHz) with device settings as follows: velocity for Doppler assessment was 30 cm/sec, two color maps (red and blue), angle of insonation was 45°, and pulse repetition frequency was 4000 kHz [23]. The gestational age was determined by eye ball orbital diameter, brain diameter, and date of ovulation [24].

### B-mode ultrasound examination

**Fig. 1:**
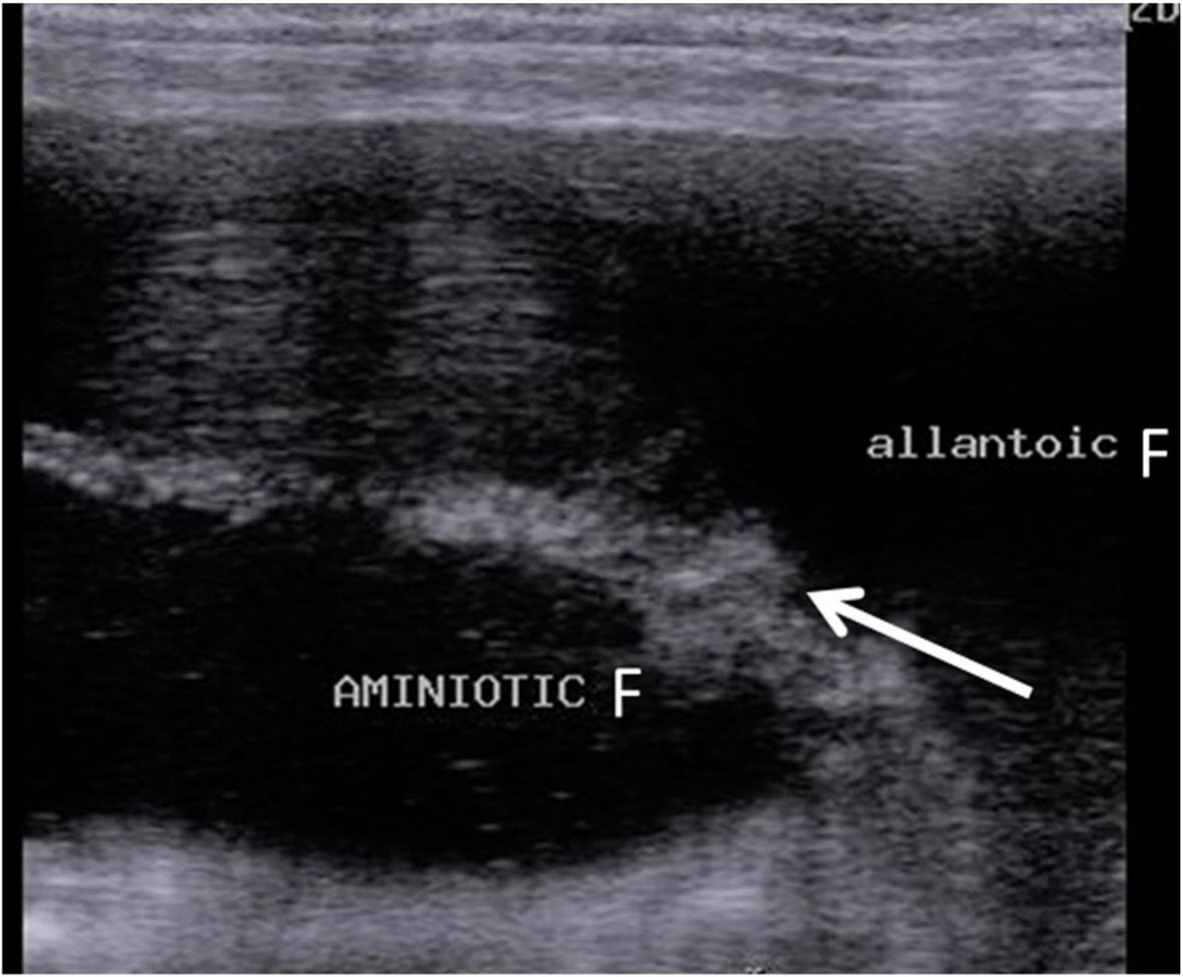
B-mode grey ultrasonogram revealed the combined thickness of both uterus and placenta (CTUP, white arrow) of pregnant she donkey with a gestation length of 200 days with presence of both amniotic and allantoic fluids.F = fluid

Ultrasound examinations were routinely performed monthly; first, a B-mode transrectal probe was activated to measure both the combined thickness of the uterus and placenta (CTUP; mm) and umbilical artery cross-sectional diameter (Umb-A. Cross sectional D; mm). For the determination of CTUP, three to five points of thickness were evaluated at the level between the middle uterine artery (MUA) and the allantoic sac as shown in Fig. 1, which was previously measured by Campos et al., [16]. While the cross-sectional umbilical artery diameter was determined by taking the maximum diameter three to five times and the average was calculated [25].

### Doppler mode ultrasound examination

**Fig. 2:**
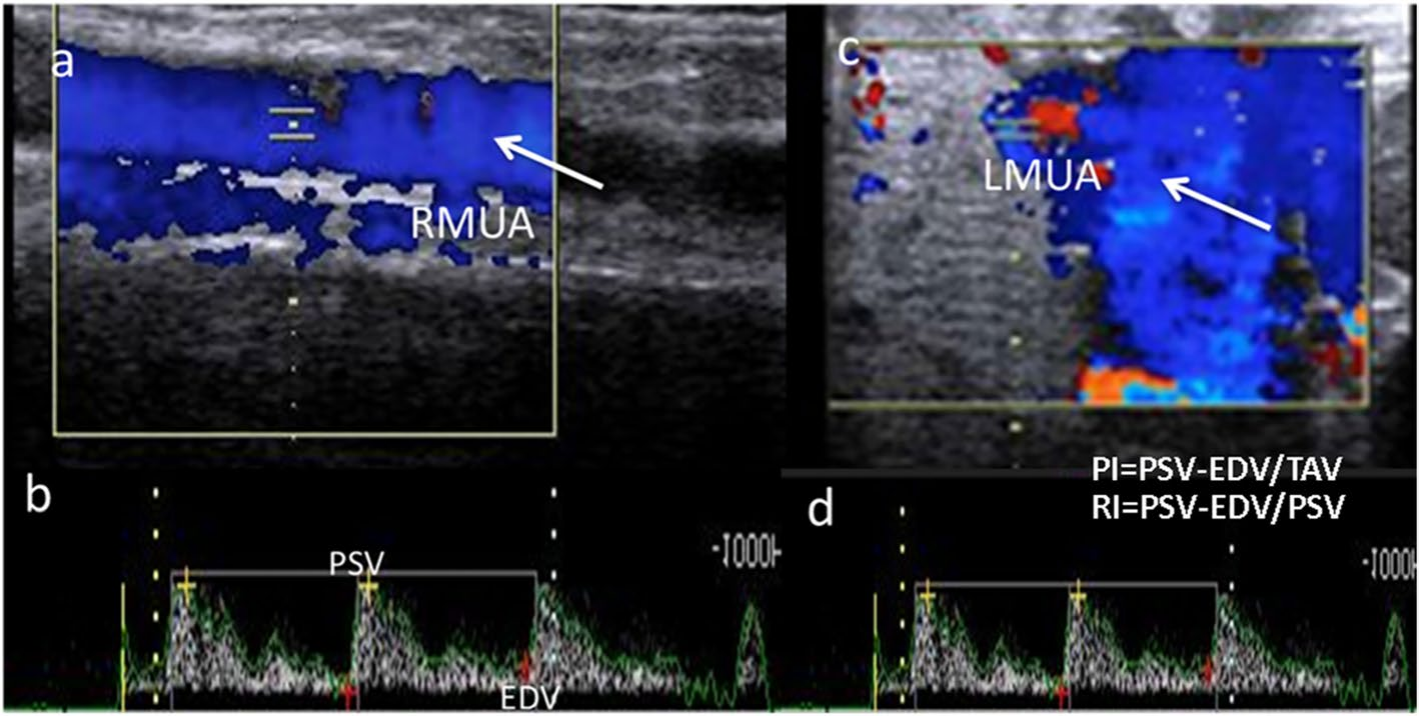
Pulsed wave and colored Doppler modes ultrasonograms revealed both right (**a**, **b**) and left (**c**, **d**) middle uterine artery of pregnant she donkey with a gestation length of 200 days with calculation of spectral Doppler indices (resistance [RI] and pulsatility indices [PI]) via both peak velocity (PSV; cm/sec) and end velocity (EDV; cm/sec).R = right, and L = left

**Fig. 3:**
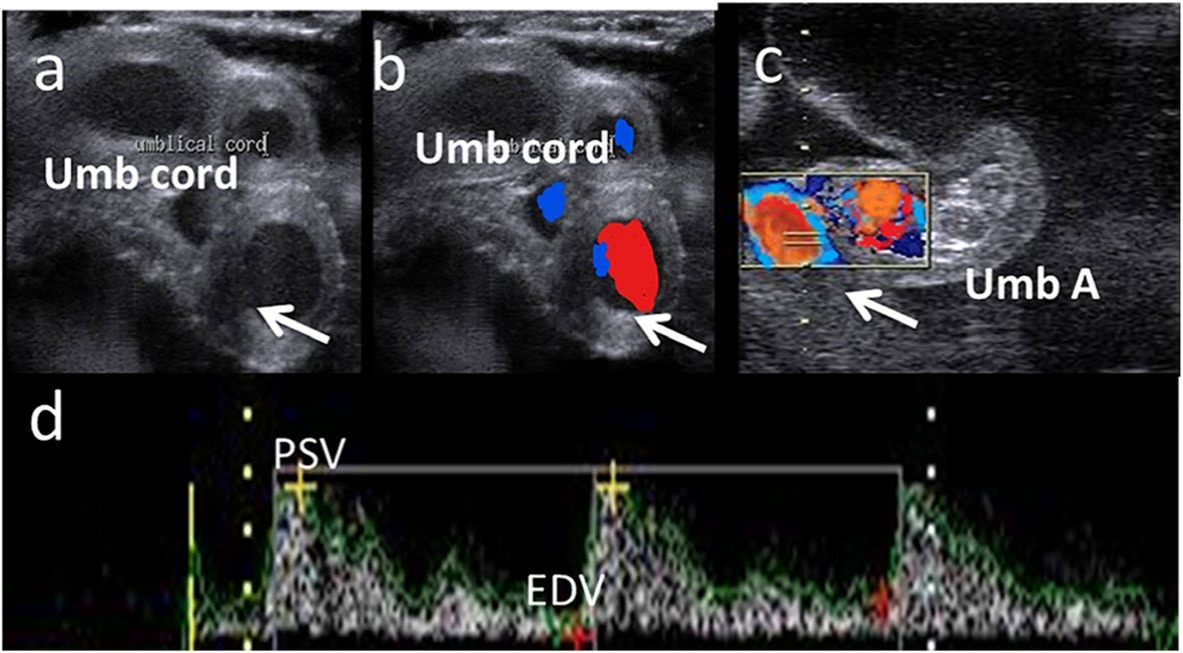
B –mode (**a**), colored (**b**) and spectral (**c**, **d**) Doppler ultrasonograms revealed umbilical cord (Umb cord)with umbilical artery (Umb A) of pregnant she donkey with a gestation length of 200 days with calculation of spectral Doppler indices resistance [RI] and pulsatility indices [PI]) via both peak velocity(PSV; cm/sec) and end velocity (EDV; cm/sec)

The pulsed wave Doppler was activated via its spectral graph of both the middle uterine (right and left) and umbilical arteries. Doppler indices expressed by resistance (RI) and pulsatility indices (PI) were automatically calculated, and the blood flow rate (bpm) was also measured.The artery was determined after identification of the aorta location and the external iliac artery as both middle uterine arteries are located at the right and left sides [26], as shown in Fig. 2.The waves were determined as three successive spectral waves in both the uterine and umbilical regions. The umbilical artery was identified at the umbilical cord [27], as shown in Fig. 3.

### Statistical analysis

All obtained data are presented as the mean ± standard error (SEM). The combined thickness of uterus and placenta (CTUP) was measured at first for the normality by using Shapiro Wilk test. Analysis of variance (ANOVA) was used to compare of the means according to gestational months and age. In pulsed wave Doppler measurement ANOVA was used also. Duncan’s range test was used to set a significant difference at *P* < 0.05.Pearson's correlation coefficients test was used to show any variation in the pregnancy months, jennies age, CTUP thickness (mm) and Doppler indices of both uterine and umbilical arteries.

## Results

The statistical analysis was significant as explained by combined thickness of both uterus and placenta (CTUP; mm) changes of the period of pregnancy and the age of animals(*P* < 0.05).Thepregnancy period and jennies ages were statistically significant (*P* < 0.01) with an interaction between both variables (*P* < 0.01). CTUP was elevated significantly within the normal range of pregnancy for all jennies at different ages from 4 to 14 years old from the 5^th^ month of gestation (Table 1; *P* < 0.05). The marked elevation in CTUP was observed in CTUP was markedly elevated in group with (9–14 years) from 5 to 9^th^ months (3.99 ± 0.01 to 5.44 ± 0.01).

**Table 1:** Combined thickness of both uterus and placenta (CTUP; mm) at different ages and different months of pregnancy in she donkeys. Data are obtained as mean ± SEM

Months	Age of jennies(Years)		
	**4–5**	**6–8**	**9–14**
**5**	3.52 ± 0.21^A.a^	3.87 ± 0.04^A,a^	3.99 ± 0.01^A,a^
**6**	3.76 ± 0.01^A.a^	4.11 ± 0.11^B,ab^	4.12 ± 0.03^B,a^
**7**	4.88 ± 0.32^AB.b^	4.41 ± 0.32^A.ab^	4.99 ± 0.05^B,ab^
**8**	5.22 ± 0.04^B.b^	4.77 ± 0.01^A,b^	5.24 ± 0.25^A,b^
**9**	5.32 ± 0.01^B,b^	4.87 ± 0.02^A,b^	5.44 ± 0.01^B,b^

Different superscript with capital are significant (*P* < 0.05) different between rows, while small superscript are significant (*P* < 0.05) different between columns

As shown in Table 2, both Doppler indices expressed by (resistance index; RI, and pulsatility index; PI) of the left (L) side middle uterine artery (MUA) differed at different jennies ages, as L. PI significantlydeclined throughout different jennies ages, but this declining trend was not observed in L. RI (*P* < 0.05).In addition; the blood flow rate (R; bpm; Fig. 2) was significantly (*P* < 0.05) elevated among different ages with 64.11 ± 0.22 for jenny aged 4–5 years, 70.12 ± 0.22 for jenny aged 6–8 years,and 75.21 ± 0.88 for jenny aged 9–14 years. However, nethier the Doppler indices nor the blood flow rate of MUA on the right side (R; bpm) showed any significant change. In Table 3, L. MUA RI and PI were examined at different gestational months from 5 to 9 months. Both RI and PI were significantly decreased from the 5^th^ to the 9^th^ month of gestation period in jenny(*P* < 0.05), but the blood flow rate of MUA was significantly elevated at different months of gestation(*P* < 0.05), while both Doppler indices and blood flow rate of MUA at the right side did not affected at different gestational months. As shown in Table 4, umbilical arteries cross-sectional diameter (Umb A. Cross sectional D; mm) was elevated in jenny at different ages, in addition, both Doppler indices were significantly declined in jenny at 9–14 years compared to other ages (*P* < 0.05),while umbilical artery (Umb A.) blood flow rate was not affected. In Table 5, umbilical arteries cross sectional diameter (Umb A. Cross-sectional D; mm) was significantly increased from the 5^th^ month till the 9^th^ months of gestation in jenny (*P* < 0.05). Both Doppler indices were significantly declined in jenny from the 5^th^ till the 9^th^ months in pregnant jenny (*P* < 0.05), in addition; umbilical blood flow rate (R; bpm) was elevated significantly in jenny at different gestational months (*P* < 0.05). There were no significant correlations between CTUP and PI (uterine and umbilical), also no significant correlation was observed between both CTUP and RI (uterine and umbilical), but a strong positive correlation (r = 0.87; *P* < 0.01) was observed between both Doppler indices in uterine and umbilical arteries.

**Table 2:** Middle uterine arteries at both sides (right and left sides) in jennies at different ages Data are obtained as mean ± SEM

Variable	Data	Age of jennies(Years)		
		**4–5**	**6–8**	**9–14**
**L. MUA PI**	Number	22	20	22
	Mean ± SEM	1.73 ± 0.01^A^	1.64 ± 0.01^AB^	1.51 ± 0.01^B^
**L. MUA RI**	Number	22	20	22
	Mean ± SEM	0.67 ± 0.01^A^	0.88 ± 0.01^B^	0.81 ± 0.02^B^
**L. MUA R(bpm)**	Number	22	20	22
	Mean ± SEM	64.11 ± 0.22^A^	70.12 ± 0.22^B^	75.21 ± 0.88^B^
**R. MUA PI**	Number	22	20	22
	Mean ± SEM	1.88 ± 0.01	1.85 ± 0.01	1.85 ± 0.01
**R. MUA RI**	Number	22	20	22
	Mean ± SEM	0.87 ± 0.02	0.85 ± 0.02	0.86 ± 0.01
**R. MUA R(bpm)**	Number	22	20	22
	Mean ± SEM	64.33 ± 2.31	66.52 ± 1.02	67.25 ± 1.11

*L* Left, *R* Right, *MUA* Middle uterine artery, *PI* Pulsatility index, *RI* Resistance index, *ipsi* ipsilateral, *contra* Contralateral, *SEM* Standard error of mean, *Number* Number of observation.Different superscripts are significant (*P* < 0.05) different between rows

**Table 3:** Middle uterine arteries at both sides (right and left side) in jennies at different gestational months Data are obtained as mean ± SEM

Variable	Data	Months of pregnancy				
		**5**	**6**	**7**	**8**	**9**
**L. MUA PI**	Number	18	17	22	20	19
	Mean ± SEM	1.76 ± 0.01^A^	1.69 ± 0.01^AB^	1.42 ± 0.01^B^	1.41 ± 0.01^C^	1.39 ± 0.01^C^
**L. MUA RI**	Number	18	17	22	20	19
	Mean ± SEM	0.68 ± 0.01^A^	0.54 ± 0.01^AB^	0.51 ± 0.02^B^	0.51 ± 0.01^B^	0.46 ± 0.01^B^
**L. MUA Rate(bpm)**	Number	18	17	22	20	19
	Mean ± SEM	66.22 ± 2.01^A^	71.02 ± 2.54^AB^	72.33 ± 2.33^AB^	76.32 ± 0.21^B^	77.52 ± 0.32^B^
**R. MUA PI**	Number	18	17	22	20	19
	Mean ± SEM	1.87 ± 0.01	1.83 ± 0.01	1.85 ± 0.01	1.86 ± 0.01	1.83 ± 0.02
**R. MUA RI**	Number	18	17	22	20	19
	Mean ± SEM	0.81 ± 0.02	0.82 ± 0.02	0.81 ± 0.01	0.82 ± 0.01	0.83 ± 0.02
**R. MUA Rate(bpm)**	Number	18	17	22	20	19
	Mean ± SEM	64.58 ± 2.31	65.44 ± 1.22	64.95 ± 2.58	66.25 ± 0.02	65.32 ± 0.01

*L* Left, *R* Right, *MUA* Middle uterine artery, *PI* Pulsatility index, *RI* Resistance index, *ipsi* ipsilateral, *contra* Contralateral, *SEM* Standard error of mean, *Number* Number of observation.Different superscripts are significant (*P* < 0.05) different between rows

**Table 4:** Umbilical arteries diameter and blood flow in jennies at different ages. Data are obtained as mean ± SEM

Variable	Data	Age of jennies(Years)		
		**4–5**	**6–8**	**9–14**
**Umb A. Cross sectional D(mm)**	Number	22	20	22
	Mean ± SEM	13.02 ± 0.33^A^	13.66 ± 0.52^AB^	14.37 ± 0.21^B^
**Umb A. PI**	Number	22	20	22
	Mean ± SEM	1.59 ± 0.01^A^	1.61 ± 0.01^A^	1.46 ± 0.01^B^
**Umb A. RI**	Number	22	20	22
	Mean ± SEM	0.67 ± 0.01^A^	0.68 ± 0.01^A^	0.51 ± 0.02^B^
**Umb A. rate**	Number	22	20	22
	Mean ± SEM	64.71 ± 1.22	68.22 ± 0.22	70.81 ± 0.88

*D* Diameter, *Umb A* Umbilical artery, *PI* Pulsatility index, *RI* Resistance index, *SEM* Standard error of mean, *Number* Number of observation.Different superscripts are significant (*P* < 0.05) different between rows

**Table 5:** Umbilical arteries diameter and blood flow in jennies at different gestational months. Data are obtained as mean ± SEM

Variable	Data	Months of pregnancy				
		**5**	**6**	**7**	**8**	**9**
**Umb A. Cross sectional D**	Number	18	17	22	20	19
	Mean ± SEM	12.02 ± 0.01^A^	12.65 ± 0.01^AB^	13.02 ± 0.01^B^	14.05 ± 0.01^C^	14.11 ± 0.01^C^
**Umb A. PI**	Number	18	17	22	20	19
	Mean ± SEM	1.54 ± 0.01^A^	1.21 ± 0.01^AB^	1.11 ± 0.02^B^	1.01 ± 0.01^B^	0.98 ± 0.01^B^
**Umb A. RI**	Number	18	17	22	20	19
	Mean ± SEM	0.77 ± 0.01^A^	0.71 ± 0.01^AB^	0.62 ± 0.01^B^	0.60 ± 0.01^B^	0.61 ± 0.02^B^
**Umb A. rate**	Number	18	17	22	20	19
	Mean ± SEM	55.31 ± 0.58^A^	59.20 ± 2.32^AB^	61.33 ± 2.22^AB^	65.24 ± 0.66^B^	66.25 ± 0.99^B^

*D* Diameter, *Umb A* Umbilical artery, *PI* Pulsatility index, *RI* Resistance index, *SEM* Standard error of mean, *Number* Number of observation.Different superscripts are significant (*P* < 0.05) different between rows

## Discussion

This is the first study to report changes in uteroplacental and umbilical blood flow patterns in pregnancy jennies. The CTUP (mm) was elevated among gestational ages, which is in accordance with some studies [15, 17, 28, 29], as all previous studies demonstrated that this elevation is within the normal values of the combined thickness and revealed that the placental tissue was functional and efficient during different stages of pregnancy [30]. Any abnormalities in CTUP thickness could adversely affect the maintenance of pregnancy and therefore lead to placental separation [31] and placentitis [32]. As fetal growth depends mainly on placental growth and development [33, 34], a study found that CTUP remained within constant levels, it did not change. Therefore, the use of transrectal ultrasonographic examination to assess CTUP is superior [15, 16, 33]. Normal values for the CTUP have been established from 5 months of gestation to 9 months in normal pregnant quarter horses [33], standard breeds [35], ponies and Arabs [36], donkeys [17], and Dutch warm blood [37]. In the current study, no correlation was observed between CTUP and PI or between CTUP and RI; similarly, a study in mares revealed that CTUP did not show any difference between placentitis-induced animals and noninfected animals [38].

Vascular expression of selected angiogenic factors has been reported in the placenta during the mid-stage of pregnancy in many species [39, 40]. Therefore, the uterine artery Doppler parameters in pregnant jenny could provide data about uterine vascularization and placental function, as previously recorded in women [41]. Our results were similar to those measured previously by Ousey et al. [42], who concluded an increase in uterine blood flow velocities, volume, and rate in the pregnant woman that was associated with an increase in gestational age to meet pregnancy requirements.

The decline in both middle uterine Doppler indices is in accordance with some studies [25, 42] that reported a marked decline in resistance and pulsatility indices (RI and PI) of the main uterine artery on the right and left sides, as this decline could reflect on the fetal growth and development of the placental microcirculation. Moreover, in cats, the uterine resistance index (RI) declined up to − 15% in abnormal cats compared to − 36% in normal ones [43]. In humans, the lowest RI occurs at approximately 24 to 25 weeks gestation and remains unchanged throughout pregnancy [44]. A simultaneous decrease in RI and PI indicates significant increases in blood flow parameters such as peak systolic, end diastolic velocities and blood flow rate [45]. The results of the current study showed changes in the uterine blood flow rate (R; bpm) during different months of pregnancy. A similar study reported an increase in Doppler velocities and blood flow rate with advancement of pregnancy that may be attributed to increased uterine arterial diameter and increased uterine size resulting from increased intrauterine fetal size [43, 46]. Other parameters were measured in our study, such as the total blood flow rate in both uterine and umbilical arteries (R; bpm), as both umbilical blood flow rates were elevated significantly within different gestational months, while within different ages, the rate was not affected. These findings are in accordance with a previous study [38] that reported an increase in total arterial blood flow rate with advancement of gestational age in the control mares (*P* = 0.0001). In this study, we reported a decline in both Doppler indices with pregnancy development; in addition, higher Doppler indices were observed in buffalo at 15 years old [19]. Finally, there was a negative correlation between uterine Doppler indices in all age jenny, as this means a marked elevation in the uterine vascular supply, especially in the left side. This finding is in agreement with a recent study in mare [18], while in contrast, another study concluded that there was no difference in blood flow between both sides [18]. This study showed a reduction in left uterine PI, especially in older jenny without any inflammatory alterations detected by ultrasound.In accordance with this study, a similar study [15] reported the same difference in the left side compared to the right one that could be related to the physiological processes of fetal and presence of the placenta with a cervical star adjacent to the cervix [2] at this side which could affect the blood flow rate in the uteroplacental region. Studies reported an increase in uteroplacental blood flow, but the uterine PI did not change either in normal or abnormal pregnant mares[3].

The significant elevation of cross-section umbilical artery diameter was in accordance with other studies [47, 48] that related to maintaining adequate blood supply to the fetus in order to get rid of any fetal growth restrictions. Similarly, changes must occur in arteries within the uterus as well, and there is increasing evidence that inadequate development of the uterine vasculature to meet pregnancy requirements may be determined primarily during the definitive placentation process and that the increase in uterine blood flow area and volume are associated with an increase in gestational age [42]. The umbilical vein blood flow area pattern was almost flat with a slightly wavy margin during all weeks of gestation, in accordance with human [49, 50] and veterinary medicines [51, 52]. Similar to our current study, gestational age related to the marked decline in umbilical artery PI has been reported [53], which reflects placental villous circulation.in addition; there was an inverse relation ship between both doppler indices and blood flow velocity [53–56], Moreover, Wladimiroff et al.[57] calculated mean RI values at different gestational ages among normal pregnant women. Therefore, noninvasive color and pulsed wave Doppler sonography have been used to evaluate uterine and umbilical blood flow changes during normal pregnancy during different gestational months at different ages, as the changes in these two parameters provide a picture of intrauterine fetal development, as previously mentioned in pregnant buffalos [58, 59], sheep [60], queens [61], and bitchs [62]. The use of Doppler spectral ultrasound in gestational observation in donkey species is recommended to prevent any pregnancy loss and abnormal placental development, with the estimation of uterine and umbilical vascularity. Therefore, it was essential to obtain data from normal healthy pregnant females to detect future jenny with high-risk pregnancy due to vascular disorders.

## Conclusion

CTUP observation is critical to determine whether this current study was performed on normal healthy pregnant jenny without any abnormal conditions throughout the gestational months. We found an elevation in both middle uterine and umbilical artery blood supply in jenny with advancement of gestational months. We demonstrated that in this species, Equus asinus, the PI and RI tend to decline within the gestational months. This decline was significant in relation to elevation in gestational age, months, CTUP and umbilical artery diameter; in addition, the age factor on the left side Doppler PI and RI was greatly suggested. This study determined the critical role of Doppler technology in this species to prevent gestational complications such as pregnancy losses and placentitis. Therefore, we recommend further studies to compare pregnant and nonpregnant healthy animals of the same donkey species.

## Data Availability

The raw data support outcomes of the present study is available by the corresponding author.
